# Immunogenetic clustering of 30 cancers

**DOI:** 10.1038/s41598-022-11366-7

**Published:** 2022-05-04

**Authors:** Lisa M. James, Apostolos P. Georgopoulos

**Affiliations:** 1 The HLA Research Group, Brain Sciences Center (11B), Department of Veterans Affairs Health Care System, Minneapolis VAHCS, One Veterans Drive, Minneapolis, MN 55417 USA; 2 Department of Neuroscience, University of Minnesota Medical School, Minneapolis, MN 55455 USA; 3 Department of Psychiatry, University of Minnesota Medical School, Minneapolis, MN 55455 USA; 4 Department of Neurology, University of Minnesota Medical School, Minneapolis, MN 55455 USA

**Keywords:** Cancer, Medical research

## Abstract

Human leukocyte antigen (HLA) genes have been implicated in cancer risk and shared heritability of different types of cancer. In this immunogenetic epidemiological study we first computed a Cancer-HLA profile for 30 cancer types characterized by the correlation between the prevalence of each cancer and the population frequency of 127 HLA alleles, and then used multidimensional scaling to evaluate the possible clustering of those Cancer-HLA associations. The results indicated the presence of three clusters, broadly reflecting digestive-skin-cervical cancers, reproductive and endocrine systems cancers, and brain and androgen-associated cancers. The clustering of cancer types documented here is discussed in terms of mechanisms underlying shared Cancer-HLA associations.

## Introduction

Cancer, a leading cause of death worldwide^1^, is associated with environmental risk factors coupled with genetic predisposition^2^. It is now well-established that genetic factors are not only a significant contributor of cancer risk but also contribute to shared heritability among different cancers^3–8^. For instance, one study evaluating the genetic correlation between six cancer types in individuals of European ancestry found significant genetic correlations of colorectal cancer with pancreatic cancer, breast cancer, and lung cancer as well as genetic correlations between lung cancer and breast cancer^6^; in contrast, they found no evidence of shared heritability between prostate cancer and the other cancers they investigated. A more recent study from the same group reported statistically significant genetic correlations between breast cancer with ovarian cancer, lung cancer, and colorectal cancer as well as lung cancer with head/neck cancer^7^. A study evaluating 13 cancer types found the strongest genetic correlations between kidney and testicular cancer, diffuse large B-cell lymphoma and osteosarcoma and chronic lymphocytic leukemia, and bladder and lung cancer^5^. Finally, a study of 18 cancer types in two large cohorts of European ancestry found several genetically associated cancer pairs including positive correlations between colon and rectal cancers; esophageal/stomach cancer and Non-Hodgkin’s lymphoma, breast, lung, and rectal cancers; bladder and breast cancers; melanoma and testicular cancer; and prostate and thyroid cancers^8^. In addition they reported four negative correlations including endometrial and testicular cancers; esophageal/stomach cancer and melanoma; lung cancer and melanoma; and Non-Hodgkin’s lymphoma and prostate cancer^8^. Moreover, there was evidence of widespread pleiotropy including 25 regions that were associated with more than 1 cancer type, 14 of which involved the Human Leukocyte Antigen (HLA) region^8^.

The HLA region of chromosome 6 codes for cell-surface proteins involved in immunosurveillance and T-cell activation aimed at elimination of tumor cells and pathogens. Specifically, Class I HLA molecules (HLA-A, -B, -C) present intracellular antigen peptides to CD8+ cytotoxic T cells to signal destruction of infected cells whereas HLA Class II molecules (HLA-DR, DQ, and DP genes) present endocytosed extracellular antigen peptides to CD4+ T cells to promote B-cell mediated antibody production and adaptive immunity. HLA has been increasingly implicated in various types of cancer^8–13^. Moreover, HLA has recently been implicated in the shared heritability across cancers with some loci evidencing unidirectional associations with cancer types and others demonstrating discordant associations with cancer types, highlighting varying associations between HLA and different types of cancer^8^.

Taken together, prior studies have identified shared genetic associations between some cancer types and implicate HLA as common genetic mechanism. HLA genes are the most highly polymorphic of the human genome. Here we take advantage of the population heterogeneity of HLA and extend prior lines of research by evaluating similarities between population-level Cancer-HLA associations involving 30 types of cancer and 127 HLA alleles. This approach captures the complex associations between HLA and cancer and permits clustering of cancers with similar HLA profiles, potentially permitting identification of common genetic mechanisms underlying clusters of cancers.

## Materials and methods

### Prevalence of 30 cancers

The population prevalence of the 30 cancers (Table 1) in 2016 was computed for each of the following 14 countries in Continental Western Europe (CWE): Austria, Belgium, Denmark, Finland, France, Germany, Greece, Italy, Netherlands, Portugal, Norway, Spain, Sweden, and Switzerland. Specifically, the total number of people with each cancer in each of the 14 CWE countries was identified from the Global Health Data Exchange^14^, a publicly available catalog of data from the Global Burden of Disease study, the most comprehensive worldwide epidemiological study of more than 350 diseases. The number of people with each cancer in each country was divided by the total population of each country in 2016^15^ and expressed as a percentage. We have previously shown that life expectancy for these countries are virtually identical^16^; therefore, life expectancy was not included in the current analyses.

**Table 1:** The 30 cancers studied in alphabetical order.

1	Bladder
2	Brain
3	Breast
4	Cervical
5	Colorectal
6	Esophageal
7	Gallbladder
8	Hodgkin
9	Kidney
10	Larynx
11	Oral
12	Liver
13	Malignant melanoma
14	Mesothelioma
15	Multiple myeloma
16	Nasopharynx
17	Neoplasms
18	Non Hodgkin
19	Non melanoma skin
20	Non melanoma basal cell
21	Non melanoma squamous cell
22	Other Pharynx
23	Ovarian
24	Pancreatic
25	Prostate
26	Stomach
27	Testicular
28	Thyroid
29	Tracheal, bronchus and lung
30	Uterine

### HLA

The frequencies of all reported HLA alleles of classical genes of Class I (A, B, C) and Class II (DPB1, DQB1, DRB1) for each of the 14 CWE countries were retrieved from the website allelefrequencies.net (Estimation of Global Allele Frequencies)^17,18^ on October 20, 2020. As we reported previously^16^, there was a total of 2746 entries of alleles from the 14 CWE countries, comprising 844 distinct alleles, i.e. alleles that occurred in at least one country. Of those, 127 alleles occurred in 9 or more countries and were used in further analyses. This criterion is somewhat arbitrary but reasonable; it was partially validated in a previous study^19^.

The distribution of those alleles (Table 2) to the HLA classes and their genes is given in Table 3.

**Table 2:** The 127 HLA alleles used and their Class, gene assignments.

Index	Allele	Class	Gene
1	A*01:01	I	A
2	A*02:01	I	A
3	A*02:05	I	A
4	A*03:01	I	A
5	A*11:01	I	A
6	A*23:01	I	A
7	A*24:02	I	A
8	A*25:01	I	A
9	A*26:01	I	A
10	A*29:01	I	A
11	A*29:02	I	A
12	A*30:01	I	A
13	A*30:02	I	A
14	A*31:01	I	A
15	A*32:01	I	A
16	A*33:01	I	A
17	A*33:03	I	A
18	A*36:01	I	A
19	A*68:01	I	A
20	A*68:02	I	A
21	B*07:02	I	B
22	B*08:01	I	B
23	B*13:02	I	B
24	B*14:01	I	B
25	B*14:02	I	B
26	B*15:01	I	B
27	B*15:17	I	B
28	B*15:18	I	B
29	B*18:01	I	B
30	B*27:02	I	B
31	B*27:05	I	B
32	B*35:01	I	B
33	B*35:02	I	B
34	B*35:03	I	B
35	B*35:08	I	B
36	B*37:01	I	B
37	B*38:01	I	B
38	B*39:01	I	B
39	B*39:06	I	B
40	B*40:01	I	B
41	B*40:02	I	B
42	B*41:01	I	B
43	B*41:02	I	B
44	B*44:02	I	B
45	B*44:03	I	B
46	B*44:05	I	B
47	B*45:01	I	B
48	B*47:01	I	B
49	B*49:01	I	B
50	B*50:01	I	B
51	B*51:01	I	B
52	B*52:01	I	B
53	B*55:01	I	B
54	B*56:01	I	B
55	B*57:01	I	B
56	B*58:01	I	B
57	C*01:02	I	C
58	C*03:03	I	C
59	C*04:01	I	C
60	C*05:01	I	C
61	C*06:02	I	C
62	C*07:01	I	C
63	C*07:02	I	C
64	C*07:04	I	C
65	C*12:02	I	C
66	C*12:03	I	C
67	C*14:02	I	C
68	C*15:02	I	C
69	C*16:01	I	C
70	DPB1*01:01	II	DPB1
71	DPB1*02:01	II	DPB1
72	DPB1*02:02	II	DPB1
73	DPB1*03:01	II	DPB1
74	DPB1*04:01	II	DPB1
75	DPB1*04:02	II	DPB1
76	DPB1*05:01	II	DPB1
77	DPB1*06:01	II	DPB1
78	DPB1*09:01	II	DPB1
79	DPB1*10:01	II	DPB1
80	DPB1*11:01	II	DPB1
81	DPB1*13:01	II	DPB1
82	DPB1*14:01	II	DPB1
83	DPB1*17:01	II	DPB1
84	DPB1*19:01	II	DPB1
85	DQB1*02:01	II	DQB1
86	DQB1*02:02	II	DQB1
87	DQB1*03:01	II	DQB1
88	DQB1*03:02	II	DQB1
89	DQB1*03:03	II	DQB1
90	DQB1*04:02	II	DQB1
91	DQB1*05:01	II	DQB1
92	DQB1*05:02	II	DQB1
93	DQB1*05:03	II	DQB1
94	DQB1*06:01	II	DQB1
95	DQB1*06:02	II	DQB1
96	DQB1*06:03	II	DQB1
97	DQB1*06:04	II	DQB1
98	DQB1*06:09	II	DQB1
99	DRB1*01:01	II	DRB1
100	DRB1*01:02	II	DRB1
101	DRB1*01:03	II	DRB1
102	DRB1*03:01	II	DRB1
103	DRB1*04:01	II	DRB1
104	DRB1*04:02	II	DRB1
105	DRB1*04:03	II	DRB1
106	DRB1*04:04	II	DRB1
107	DRB1*04:05	II	DRB1
108	DRB1*04:07	II	DRB1
109	DRB1*04:08	II	DRB1
110	DRB1*07:01	II	DRB1
111	DRB1*08:01	II	DRB1
112	DRB1*08:03	II	DRB1
113	DRB1*09:01	II	DRB1
114	DRB1*10:01	II	DRB1
115	DRB1*11:01	II	DRB1
116	DRB1*11:02	II	DRB1
117	DRB1*11:03	II	DRB1
118	DRB1*11:04	II	DRB1
119	DRB1*12:01	II	DRB1
120	DRB1*13:01	II	DRB1
121	DRB1*13:02	II	DRB1
122	DRB1*13:03	II	DRB1
123	DRB1*13:05	II	DRB1
124	DRB1*14:01	II	DRB1
125	DRB1*15:01	II	DRB1
126	DRB1*15:02	II	DRB1
127	DRB1*16:01	II	DRB1

**Table 3:** Distribution of 127 HLA alleles analyzed to Class and Genes.

Gene	Class I (N = 69 alleles)			Class II (N = 58 alleles)		
	A	B	C	DPB1	DQB1	DRB1
Count	20	36	13	15	14	29

### Data analysis

Statistical analyses were performed using the IBM−SPSS package (IBM SPSS Statistics for Windows, Version 27.0, 64-bit edition. Armonk, NY: IBM Corp; 2019) and Intel FORTRAN (Microsoft Visual Studio Community 2019, Version 16.7.5; Intel FORTRAN Compiler 2021).

### Cancer-HLA profiles and protection/susceptibility estimates

HLA profiles for each cancer were derived by computing, first, the Pearson correlation coefficient, $r$, between the prevalence of a cancer and the population frequency of an allele, and then its Fisher z-transform, $r{\prime }$, to normalize its distribution:

1 $${\text{HLA-Cancer}}\;{\text{Protection/Susceptibility}}\;\left( {P/S} \right)\;{\text{estimate}}:r^{\prime} = {\text{atanh}}\left( r \right)$$

Negative P/S estimates indicate a protective association (“protective” alleles), whereas positive P/S estimates indicate a susceptibility association (“susceptibility” alleles). Thus 30 Cancer-HLA profiles were computed, each consisting of 127 values of $r\prime$. These data were tabulated in a 127 allele (rows) × 30 cancers (columns) matrix (“Cancer-HLA” matrix).

### Multidimensional scaling (MDS)

A MDS analysis of the Cancer-HLA matrix was performed using the ALSCAL procedure of the IBM-SPSS statistical package (version 27). More specifically, we used (a) the individual differences (weighted) Euclidean distance model, where each HLA allele contributed separately to the solution, (b) Euclidean distance as the distance measure, (c) ordinal level of measurement, (d) solution in 2 dimensions, and (e) the default criteria for convergence: S-stress = 0.001, minimum s-stress value = 0.005, maximum iterations = 30.

### Spatial analysis of the MDS map: test for pure randomness

The MDS analysis yielded a 4 × 4 (arbitrary units; area = 16) 2-dimensional cancer configuration map, where each cancer type occupied a point in the map based on its X–Y (Dimension 1 and 2) coordinates. The initial step in the spatial analysis of the distribution of cancer types in this map was to test the null hypothesis that the spatial distribution of the 30 cancer types was random. For that purpose, we used the distance to nearest neighbor measure of Clark and Evans^20^, as follows. Given N = 30 points (cancer types), we computed 30 distances to nearest neighbor, ${\text{g}}_{{\text{A}}}^{k}$, one for each $k$th X–Y point, and obtained its average ${\overline{\text{g}}}_{{\text{A}}}$:

2 $${\overline{\text{g}}}_{{\text{A}}} = \frac{1}{N}\mathop \sum \limits_{k}^{k = 1, N} {\text{g}}_{{\text{A}}}^{k}$$

Next, we computed the density, $\rho$, of the observed distribution expressed as the number of points (N = 30) per unit of area (total area = 4 × 4 = 16):

3 $$\rho = \frac{N}{{{\text{area}} }}$$

The mean distance to nearest neighbor expected in an infinitely large random distribution of density $\rho$ is given by

4 $${\overline{\text{g}}}_{{\text{E}}} = \frac{1}{2\sqrt \rho }$$

With a standard error of

5 $$\sigma_{{{\overline{\text{g}}}_{{\text{E}}} }} = \frac{0.26136}{{\sqrt {N\rho } }}$$

The ratio $$G$$

6 $$G = \frac{{{\overline{\text{g}}}_{A} }}{{{\overline{\text{g}}}_{E} }}$$

can be used as a measure of the degree to which the observed distribution approaches or departs from random expectation. In a random distribution, $G = 1$, whereas under conditions of maximum aggregation, $G = 0$. Finally, the key test statistic $c$ for testing the null hypothesis of pure randomness is given by

7 $$c = \frac{{{\overline{\text{g}}}_{{{A}}} - {\overline{\text{g}}}_{{{E}}} }}{{\sigma_{{{\overline{\text{g}}}_{{{E}}} }} }}$$

where $c$ is the standard deviate of the normal curve.

### Spatial analysis of the MDS map: clustering of cancer types

We performed a K-means clustering analysis of the location of cancer types in the MDS map in order to identify possible clusters using the iterate-and-classify method of the K-means Cluster Analysis procedure of the IBM-SPSS statistical package (version 27).

### Ethical approval

This article does not contain any studies with human participants performed by any of the authors.

## Results

**Figure 1:**
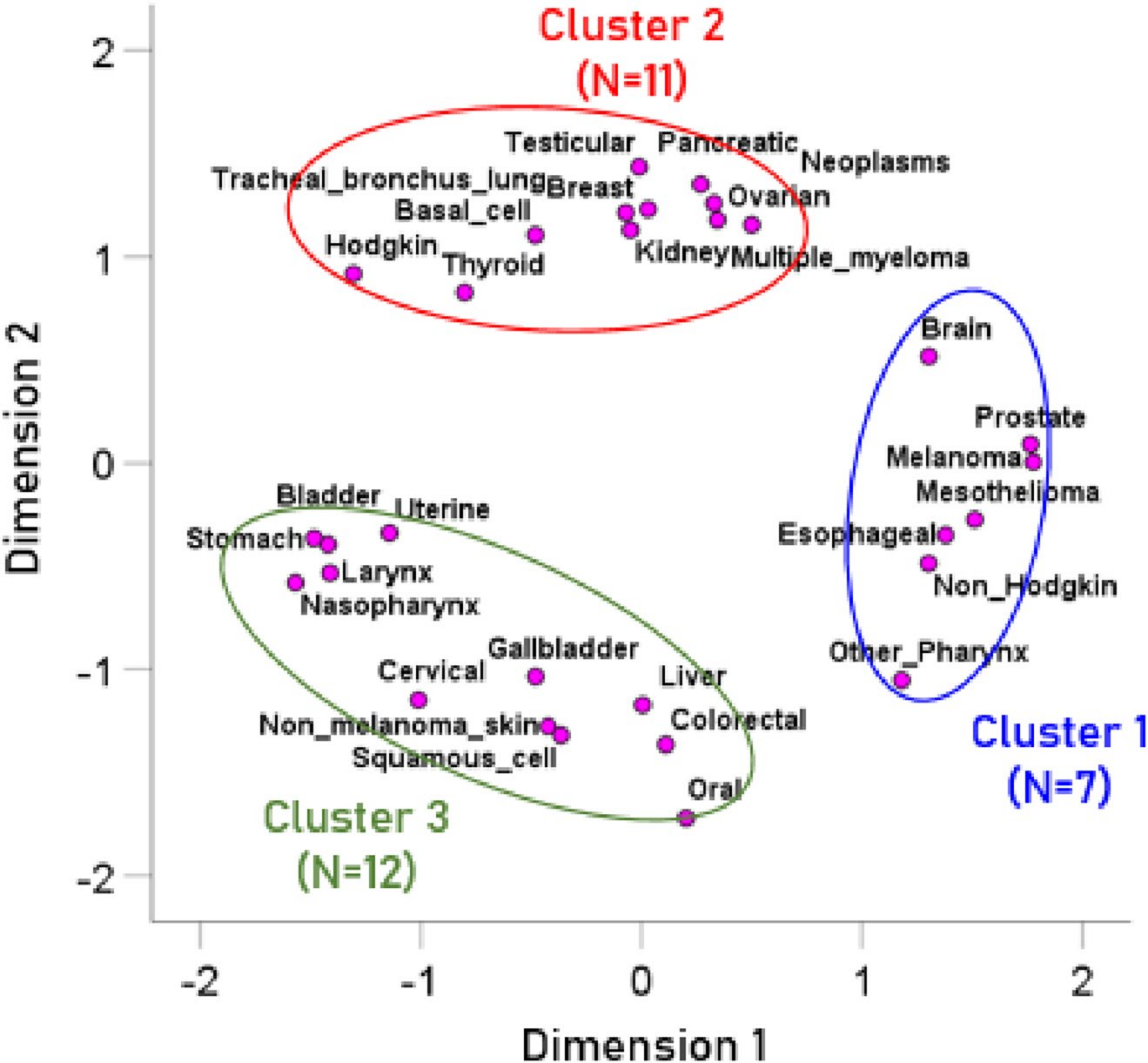
Multidimensional scaling (MDS) configuration map of the 30 cancer types with color-coded clusters of cancer types. Each point denotes the location of a cancer type within the 2-dimensional MDS configuration space. The clusters were derived by applying a K-means clustering algorithm on the x–y MDS coordinates of the cancer types. The distance between two points (cancer types) is a measure of similarity of the immunogenetic profiles of two cancers, where each such profile consisted of 127 protection/susceptibility (P/S) HLA estimates for each cancer type.

The MDS cancer type configuration map is shown in Fig. 1 and the X–Y coordinates of the 30 cancer types are given in Table 4.

**Table 4:** The X–Y coordinates of the 30 cancers in the MDS cancer types configuration map of Fig. 1.

	Cancer type	X-coordinate (Dimension 1)	Y-coordinate (Dimension 2)
1	Bladder	− 1.4208	− 0.3948
2	Brain	1.3048	0.5165
3	Breast	0.0306	1.2308
4	Cervical	− 1.0103	− 1.1487
5	Colorectal	0.1113	− 1.3644
6	Esophageal	1.38	− 0.349
7	Gallbladder	− 0.4799	− 1.0351
8	Hodgkin	− 1.306	0.9169
9	Kidney	− 0.0487	1.1292
10	Larynx	− 1.4115	− 0.5334
11	Oral	0.2041	− 1.7215
12	Liver	0.0063	− 1.1726
13	Malignant melanoma	1.7777	0.0053
14	Mesothelioma	1.5125	− 0.2735
15	Multiple myeloma	0.3451	1.1777
16	Nasopharynx	− 1.569	− 0.5803
17	Neoplasms	0.3296	1.2584
18	Non Hodgkin	1.3038	− 0.4867
19	Non melanoma skin	− 0.4234	− 1.2767
20	Non melanoma basal cell	− 0.4808	1.105
21	Non melanoma squamous cell	− 0.3636	− 1.3192
22	Other Pharynx	1.1802	− 1.0523
23	Ovarian	0.5011	1.1547
24	Pancreatic	0.2691	1.351
25	Prostate	1.7653	0.0911
26	Stomach	− 1.4845	− 0.3648
27	Testicular	− 0.0097	1.4353
28	Thyroid	− 0.8011	0.827
29	Tracheal, bronchus and lung	− 0.0697	1.2135
30	Uterine	− 1.1426	− 0.3393

### Analysis of spatial distribution of the MDS map of 30 cancer types: test for pure randomness

The following values were obtained:

9 $${\overline{\text{g}}}_{A} = 0.009456$$

10 $$\rho = \frac{30 }{{16}} = 1.875$$

11 $${\overline{\text{g}}}_{{\text{E}}} = \frac{1}{2\sqrt \rho } = 0.36515$$

12 $$G = \frac{{{\overline{\text{g}}}_{A} }}{{{\overline{\text{g}}}_{E} }} = 0.0259$$

13 $$\sigma_{{{\overline{\text{g}}}_{{\text{E}}} }} = 0.03485$$

14 $$c = \frac{{{\overline{\text{g}}}_{{{A}}} - {\overline{\text{g}}}_{{{E}}} }}{{\sigma_{{{\overline{\text{g}}}_{{{E}}} }} }} = 10.21\;\left( {P < 0.001} \right)$$

The high value of normal deviate $c$ (Eq. 14), and the corresponding very low probability value, reject the null hypothesis of pure randomness of the distribution of the 30 cancer types in the MDS configuration map (Fig. 1).

### Spatial clustering of cancer types

Inspection of Fig. 1 suggests the existence of 3 main clusters. (Interestingly, this is the same number of clusters predicted by the measure of Carlis and Bruso^21^). Therefore, we performed a K-means clustering analysis which assigned the 30 cancer types to 3 clusters (red, green and blue ellipses in Fig. 1). Cluster 1 (blue, Fig. 1) comprised 7 cancers of which melanoma and prostate cancer were tightly clustered; brain cancer and non-Hodgkin’s lymphoma were also included in this cluster. Cluster 2 (red, Fig. 1) comprised 11 cancers including several reproductive system cancers as well as some cancers involving the endocrine system. Here, breast, kidney, and tracheal-bronchus-lung cancers are tightly clustered as are pancreatic, ovarian, and neoplasms. Finally, cluster 3 (green, Fig. 1) comprised 12 cancer including several cancers of the digestive system, cervical cancer, and skin cancers.

### Immunogenetic associations between specific cancers

**Figure 2:**
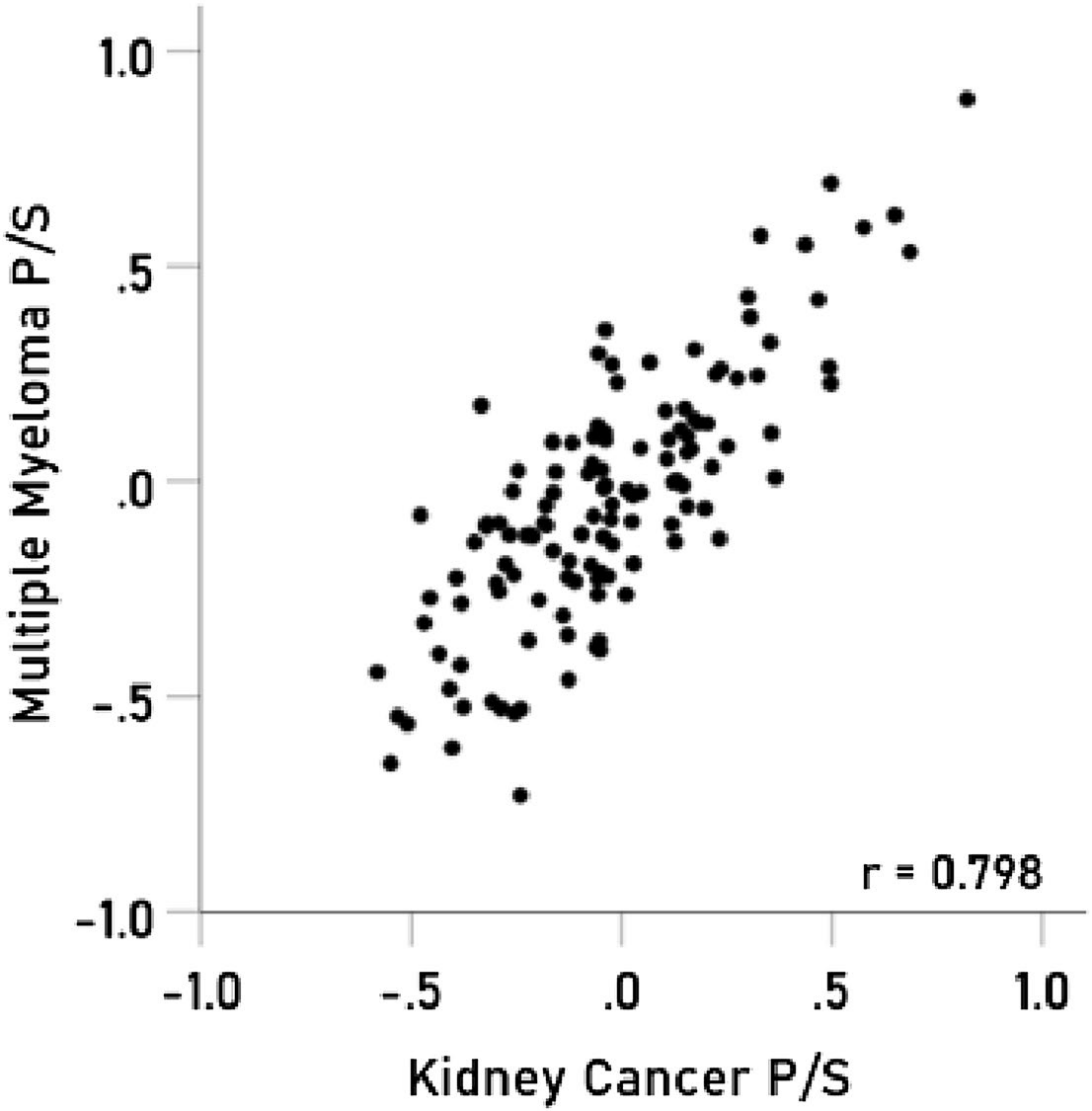
Immunogenetic P/S estimates for multiple myeloma are plotted against those for kidney cancer. r is the Pearson correlation coefficient; *P* < 0.001; N = 127.

**Figure 3:**
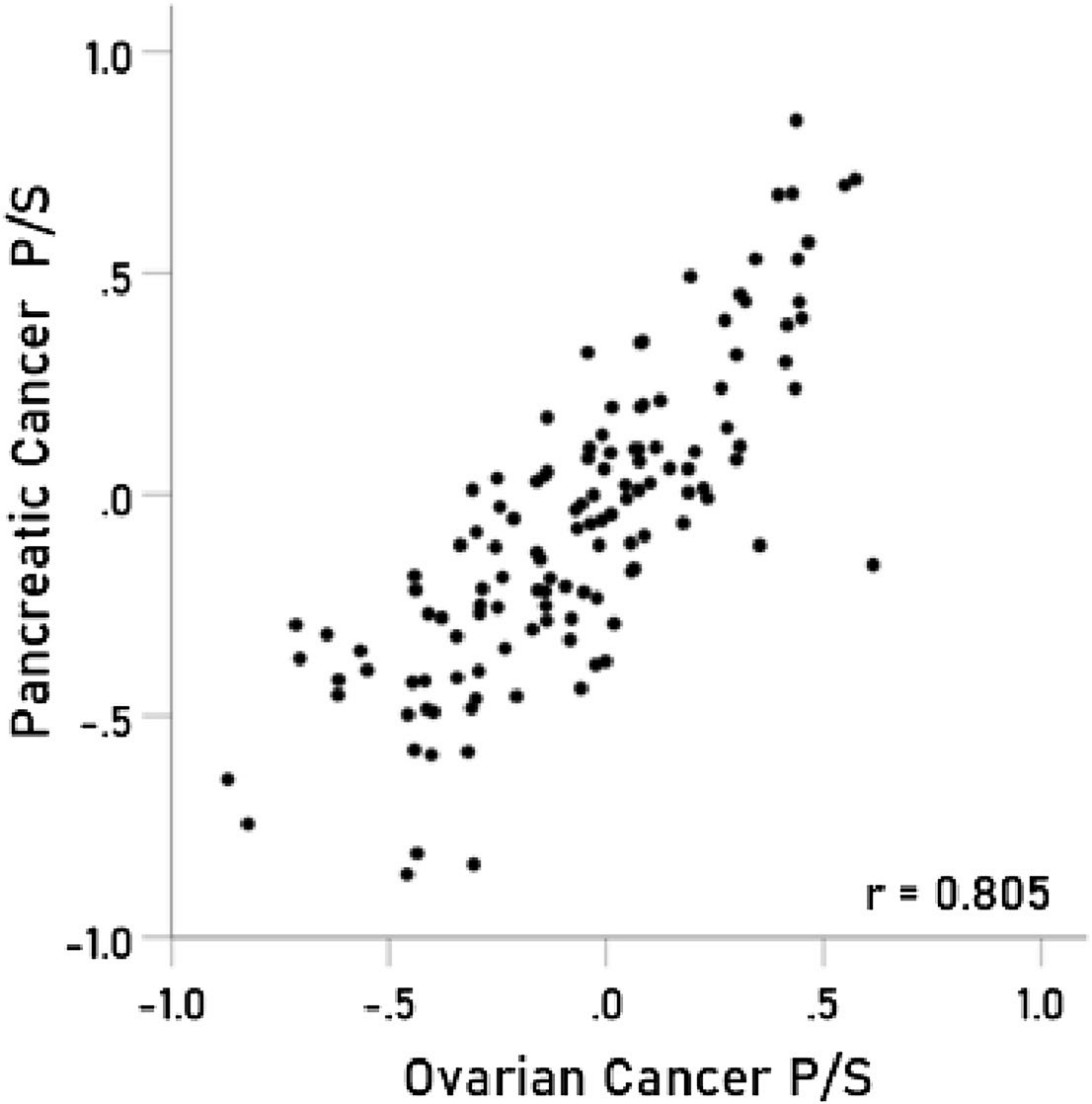
Immunogenetic P/S estimates for pancreatic cancer are plotted against those for ovarian cancer. *P* < 0.001; N = 127.

**Figure 4:**
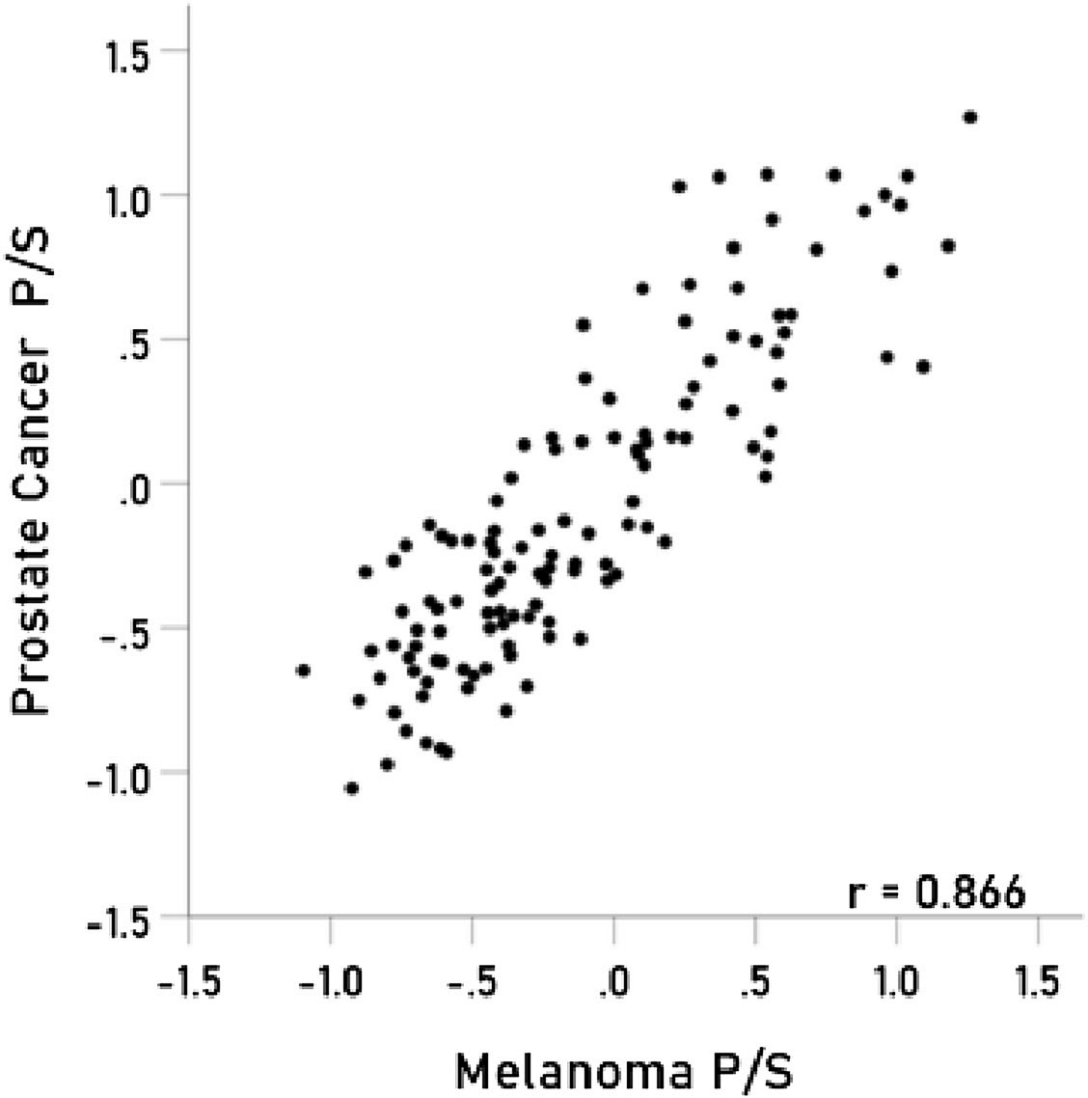
Immunogenetic P/S estimates for prostate cancer are plotted against those for melanoma. *P* < 0.001; N = 127.

**Figure 5:**
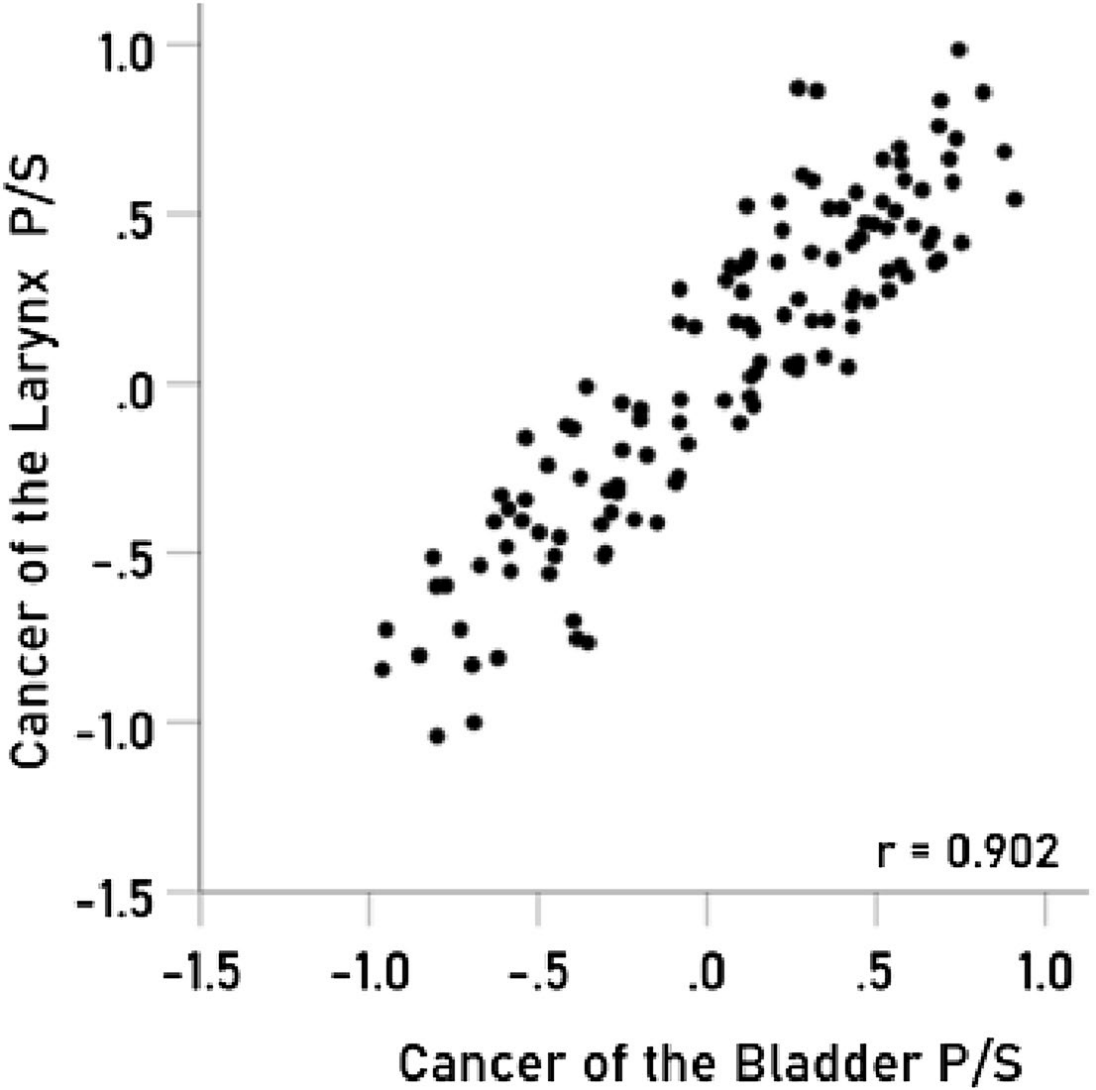
Immunogenetic P/S estimates for the cancer of larynx cancer are plotted against those for the cancer of bladder. *P* < 0.001; N = 127.

**Figure 6:**
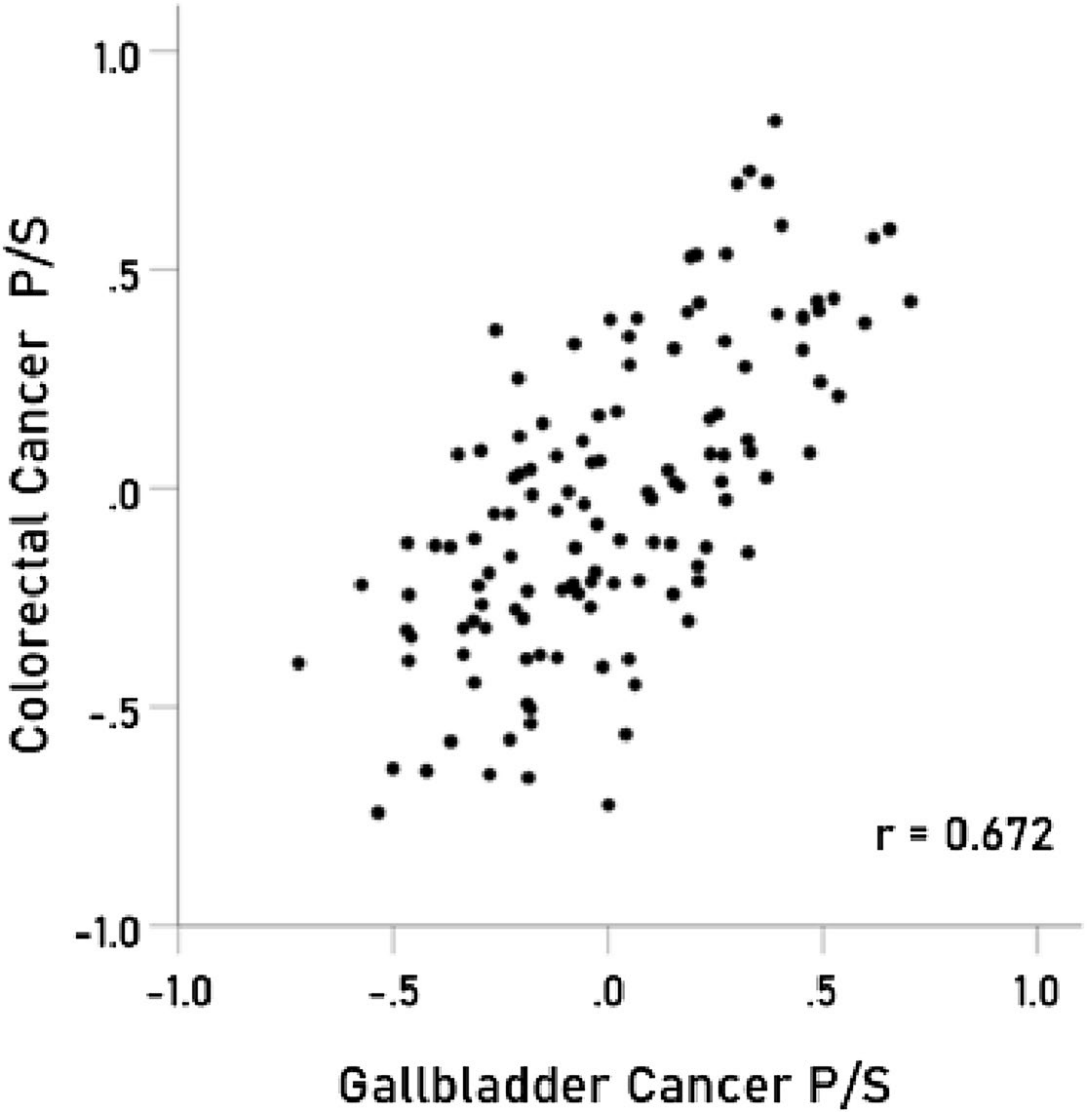
Immunogenetic P/S estimates for colorectal cancer are plotted against those for the cancer of gallbladder. *P* < 0.001; N = 127.

**Figure 7:**
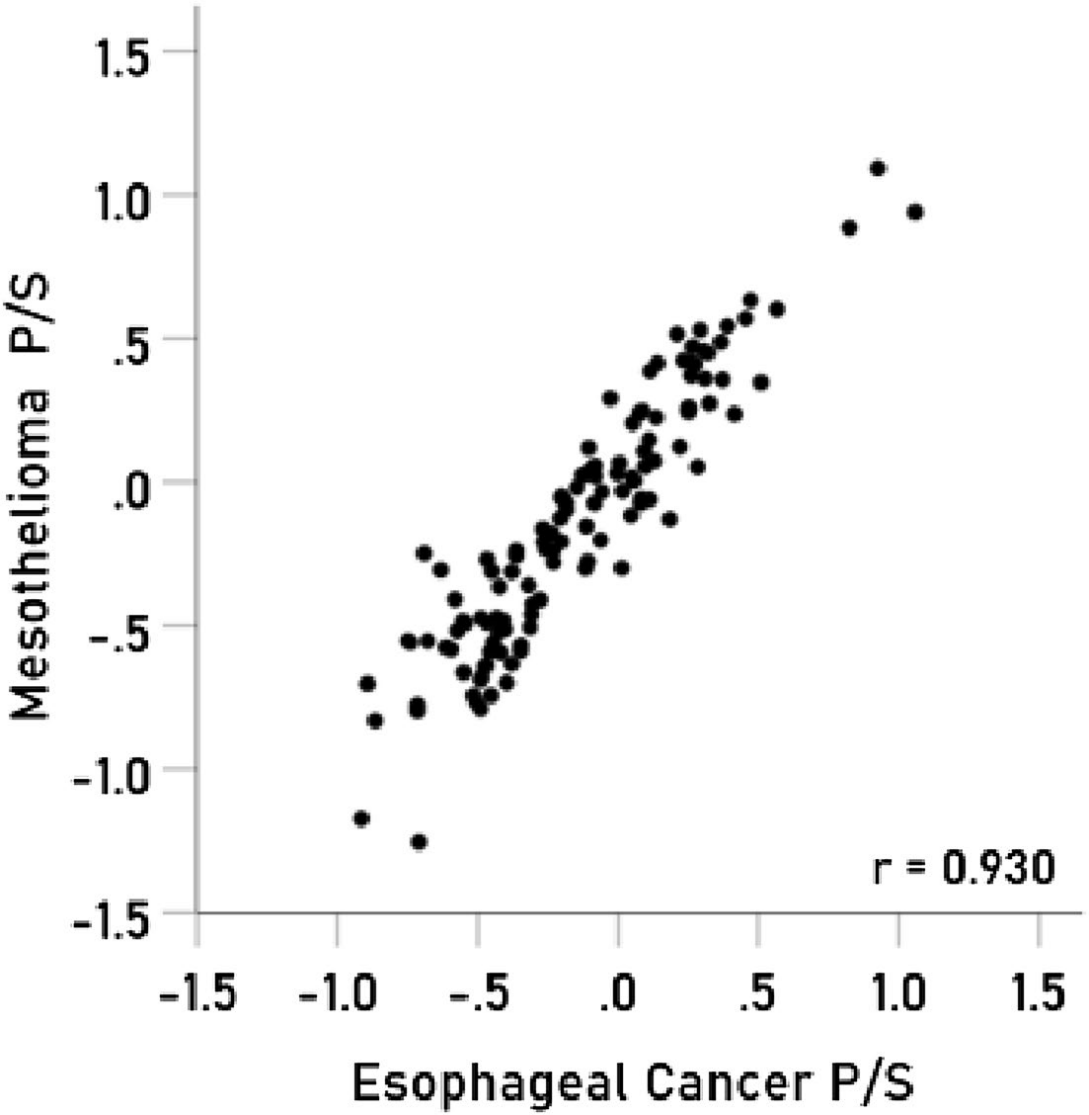
Immunogenetic P/S estimates for mesothelioma are plotted against those for esophageal cancer. *P* < 0.001; N = 127.

**Figure 8:**
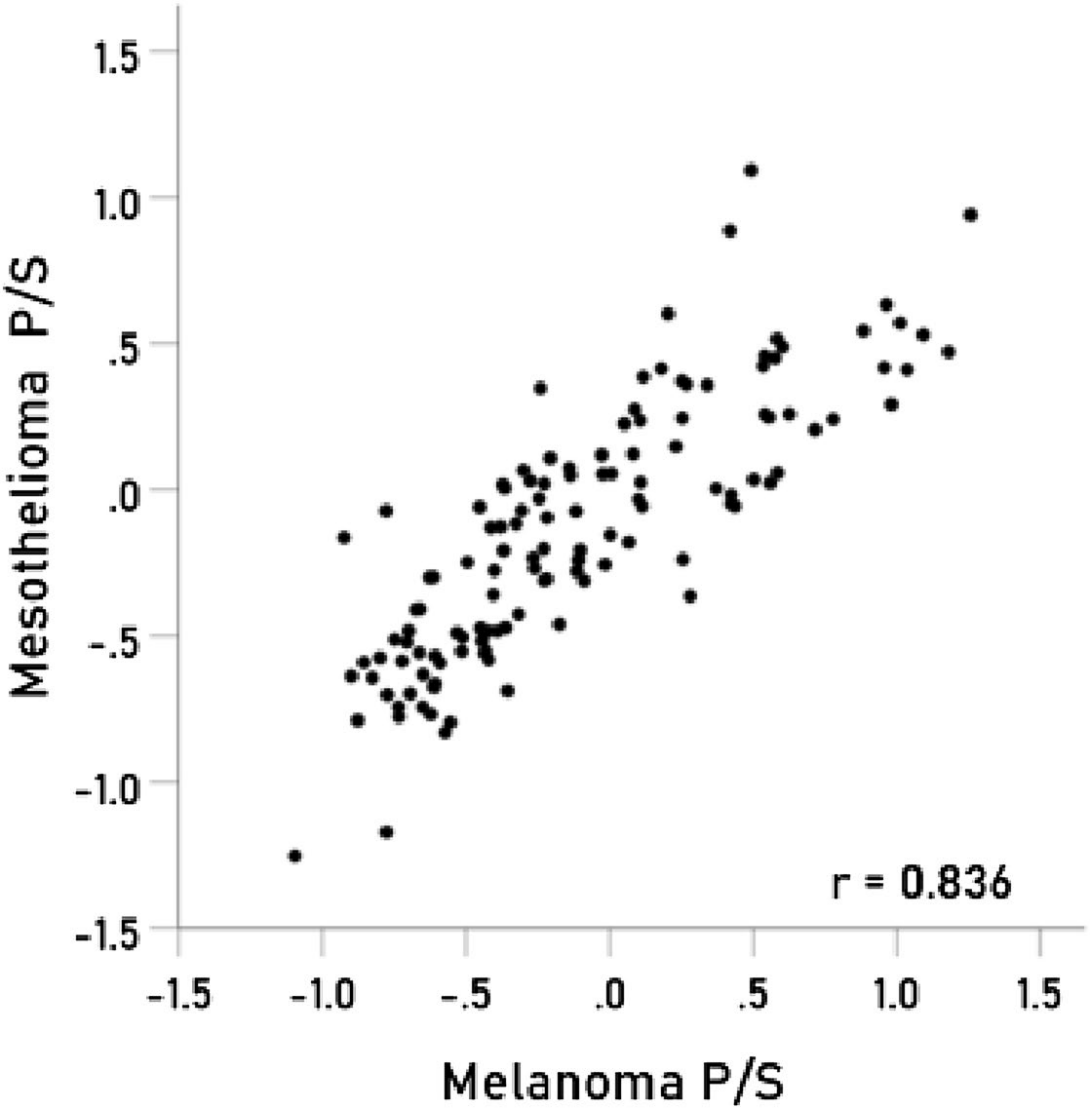
Immunogenetic P/S estimates for mesothelioma are plotted against those for melanoma. P < 0.001; N = 127.

The results above are based on associations between immunogenetic HLA-Cancer profiles of specific cancer types. It would be important to cross-validate this approach by comparing associations above with known associations between cancer types occurring in the same individual. To that end, we used 4 pairs of cancers with significant probabilities of co-occurrence in the same individuals and computed the correlations between their immunogenetic HLA-Cancer profiles to test the prediction that those correlations would be positive, high, and statistically significant, if, indeed, the immunogenetic predictions are congruent with the documented co-occurrence. The 4 pairs of cancers included (1) multiple myeloma and kidney cancer^22–25^ (Fig. 2), (2) pancreatic and ovarian cancers^26–28^ (Fig. 3), (3) prostate cancer and melanoma^29^ (Fig. 4), and (4) cancer of the bladder and larynx^30^ (Fig. 5). We also analyzed two additional cancer pairs for which common causes have been postulated but for which we could not find cases documenting their co-occurrence. One is the gallbladder-colorectal cancer pair (Fig. 6) for which gallstones have been implicated^31–33^, and the other is the mesothelioma-esophageal cancer pair (Fig. 7) for which exposure to asbestos has been implicated^34,35^. It can be seen that the immunogenetic HLA profiles of all these 6 cancer pairs were positively and highly significantly associated (*P* < 0.001 for all correlations), attesting to the congruence of existing data from other studies. Finally, the immunogenetic association between mesothelioma and melanoma P/S is illustrated in Fig. 8.

## Discussion

Here we used multidimensional scaling^36^ to identify clusters of cancers based on the population-based HLA profile of each cancer in 14 countries in Continental Western Europe. The findings, which indicate the presence of three clusters derived from the immunogenetic HLA-cancer associations, partially overlap with prior studies documenting shared genetic associations for different cancers^3–8^ and provide novel insights regarding potential shared mechanisms underlying different types of cancers. Remarkably, the immunogenetic associations derived here were congruent with known associations between the co-occurrence (or the significant probability of co-occurrence) of certain types of cancers, thus lending credit to our immunogenetic approach.

The three clusters derived (Fig. 1) are based on similarities of the HLA profiles among cancer types: the more similar the HLA profile between two cancers, the closer these cancers will be in the map of Fig. 1. Given the role of HLA in antigen elimination, it can be inferred that a given cluster may reflect similar HLA binding to shared neoantigens^37^, epitopes common to different antigens, and/or viral oncoproteins^38,39^ associated with several types of cancer within a cluster. Additional research is warranted to identify the specific factors linking cancers within each cluster and those that distinguish different clusters.

Several associations documented here overlap with those of previous studies and several others are novel. Consistent with the clustering observed in our study, previous studies have documented genetic associations between several types of cancer including breast cancer and lung cancer^6,7^, breast cancer and ovarian cancer^7^, kidney and testicular cancers^5^, and esophageal cancer and Non-Hodgkin’s lymphoma^8^. The present findings provide additional support regarding shared genetic associations between these cancer types and specifically point to highly similar HLA as partially driving those associations. Prior studies evaluating genetic associations between different types of cancer have been limited by inclusion of relatively few types of cancer and very few studies have specifically evaluated HLA with regard to genetic associations. Here we included 30 different cancers and 127 different HLA alleles permitting identification of overlapping cancer-HLA associations that have not previously been reported to our knowledge. For example, we found that prostate cancer and melanoma are very tightly clustered, implicating HLA in that shared risk. Prior studies have documented a possible link between melanoma risk and prostate cancer risk, purportedly associated with androgen-dependence^40,41^. Esophageal cancer and non-Hodgkin’s lymphoma, which were clustered with melanoma and prostate cancer here, have also been linked to androgens^42,43^. Though intriguing, a mechanism linking HLA to androgen-associated cancers remains to be elucidated.

Overall, the findings of the present study document similarities and differences among several types of cancer based on HLA associations in Continental Western European Countries. It should be noted, however, that the findings may not generalize to other populations since HLA varies across populations^44,45^. For instance, the genetic architecture of lung cancer has been shown to vary by population and environmental exposures^5^. Furthermore, although environmental factors play a role in risk of cancers^2,3^, we solely focused on the influence of HLA and did not evaluate the influence of environmental exposures in the present study. Nonetheless, the results provide compelling evidence regarding the influence of HLA on the population clustering of different types of cancer and suggest that additional research evaluating Cancer-HLA associations is warranted. As discussed above, HLA relates to antigens, and, as such, HLA-based measures and associations are necessarily discussed in the context of antigen presentation and processing by HLA. Given the diversity of proteins produced by tumor cells, the concomitant degradation of host proteins, and the added potential viral proteins implicated in several cancers, it is not surprising that the HLA-Cancer immunogenetic profiles provide a broad spectrum of cancer protection/susceptibility estimates reflecting the footprint, so to speak, of a specific cancer with regard to the antigens related to the cancer. In that sense, the observed associations and clustering described here furnish a framework within which a 127-dimensional HLA allele map is effectively reduced to a 2-dimensional map by multidimensional scaling that allows for a simplified investigation of immune-related associations among cancer types. Since the origin of the various antigens involved can be very diverse, as mentioned above, the associations found among the various cancer types are more holistic in nature, and their interpretation rests on information stemming from considerations regarding the antigens themselves. For example, a major part of the co-occurrence of pancreatic and ovarian cancers stems from the presence of BRCA genes in individuals susceptible to both cancers but their corresponding significant immunogenetic association indicates that the spectrum of antigens related to those cancers is very similar in those cancers despite the fact that they affect very different cells (pancreas vs. ovaries). Similar considerations hold for the more tenuous attribution of the mesothelioma/esophageal cancer association to asbestos and of the gallbladder/colorectal cancer association to gallstones. A recent study linking mesothelioma to melanoma^46^ via BAP1 gene highlights the predictive power of our immunogenetic approach which showed a high correlation between mesothelioma and melanoma HLA P/S estimates (Fig. 8).

In summary, the immunogenetic HLA-Cancer association approach is a promising new tool in identifying associations between cancer types which may be due to various causes (genetic, environmental, viral, etc.) but which produce a spectrum of antigens that engage in a similar way the HLA system. Given that the success of immune blockade immunotherapy for cancer partly depends on the HLA genetic makeup of the patient^11–13^, the HLA-based cancer associations we report here could be useful in informing immunotherapy across various cancer types.

## Data Availability

All data used were retrieved from freely accessible websites, as mentioned in Methods, and, as such, are publicly and freely available.
